# Generation of non-viral, transgene-free hepatocyte like cells with *piggyBac* transposon

**DOI:** 10.1038/srep44498

**Published:** 2017-03-15

**Authors:** Hokahiro Katayama, Kentaro Yasuchika, Yuya Miyauchi, Hidenobu Kojima, Ryoya Yamaoka, Takayuki Kawai, Elena Yukie Yoshitoshi, Satoshi Ogiso, Sadahiko Kita, Katsutaro Yasuda, Naoya Sasaki, Ken Fukumitsu, Junji Komori, Takamichi Ishii, Shinji Uemoto

**Affiliations:** 1 Department of Surgery, Graduate School of Medicine, Kyoto University, Kyoto, Japan; 2 Center for iPS Cell Research and Application (CiRA), Kyoto University, Kyoto, Japan

## Abstract

Somatic cells can be reprogrammed to induced hepatocyte-like cells (iHeps) by overexpressing certain defined factors in direct reprogramming techniques. Of the various methods to deliver genes into cells, typically used genome-integrating viral vectors are associated with integration-related adverse events such as mutagenesis, whereas non-integrating viral vectors have low efficiency, making viral vectors unsuitable for clinical application. Therefore, we focused on developing a transposon system to establish a non-viral reprogramming method. Transposons are unique DNA elements that can be integrated into and removed from chromosomes. *PiggyBac*, a type of transposon, has high transduction efficiency and cargo capacity, and the integrated transgene can be precisely excised in the presence of transposase. This feature enables the *piggyBac* vector to achieve efficient transgene expression and a transgene-free state, thus making it a promising method for cell reprogramming. Here, we attempted to utilize the *piggyBac* transposon system to generate iHeps by integrating a transgene consisting of *Hnf4a* and *Foxa3*, and successfully obtained functional iHeps. We then demonstrated removal of the transgene to obtain transgene-free iHeps, which still maintained hepatocyte functions. This non-viral, transgene-free reprogramming method using the *piggyBac* vector may facilitate clinical applications of iHeps in upcoming cell therapy.

Therapy for lethal liver failure has mostly been limited to liver transplantation up to the present date, and the shortage of donor livers is a serious problem. Recently, cell transplantation is considered to be an alternative therapeutic choice. Although the leading cell-source candidate should be hepatocytes, it remains difficult to establish stable procedures for their isolation, preservation, and supply^1^,^2^,^3^,^4^. To overcome this hurdle, hepatocyte-like cells generated from the patient’s own somatic cells are expected to be an attractive alternative. Experimental procedures have been reported for differentiation of other cell types into hepatocyte-like cells, such as mesenchymal stem cells (MSCs)^5^,^6^,^7^, embryonic stem cells (ESCs)^8^,^9^, and induced pluripotent stem cells (iPSCs)^10^,^11^, under specific culture conditions. Since 2011, when Suzuki *et al*. and Huang *et al*. established a direct reprogramming technique to reprogram mouse fibroblasts to induced-hepatocyte like cells (iHeps) by overexpressing defined factors^12^,^13^, research in this field has proceeded rapidly.

Viral vectors have commonly been used as gene delivery tools because of their capability to transduce genes efficiently. However, although integrative vectors, such as retro- or lentivirus, have been utilized in many reprogramming trials for their strong ability to integrate and drive the expression of genes of interest, they present risks of genome mutagenesis and genotoxicity^14^,^15^ resulting from random genome integration. In contrast, non-integrative vectors, such as adenovirus, only provide transient gene expression and exhibit lower reprogramming effciency^16^,^17^. Therefore, given that viral vectors require special care in handling, non-viral vectors are preferable for generating clinically applicable iHeps.

Recently, non-viral methods to generate iHeps using synthetic mRNA^18^, microRNA^19^, or episomal vectors^20^ have been reported; however, these methods have low reprogramming efficiency and the procedures are complicated. In the present study, we focused on a *piggyBac* transposon system to establish a non-viral, transgene-free reprogramming method. Transposons are unique DNA segments that were discovered in animals and plants as reversibly “mobile elements” in the genome^21^. In the presence of transposase, transposons are excised from a region on the host genome and integrated into another region of the genome. *PiggyBac*, a transposon discovered in the cabbage looper moth *Trichoplusia ni*^22^, possesses a large cargo size and high transposition efficiency in mammalian cells^23^,^24^. Additionally, the excision of the transposon segment is precise and leaves no footprint on the host genome in which the transgene was integrated, thereby reducing the risk related to integration and making *piggyBac* a suitable vector for cell reprogramming^25^,^26^. In the present study, we attempted to generate iHeps from mouse MSCs by using *piggyBac*-based direct reprogramming to overexpress a transgene, and then removed the transgene to generate transgene-free iHeps. With this strategy, we successfully obtained functional transgene-free iHeps. This approach has the potential to advance iHep generation toward clinical application.

## Results

### Generating factor-integrated iHeps from mouse MSCs

**Figure 1:**
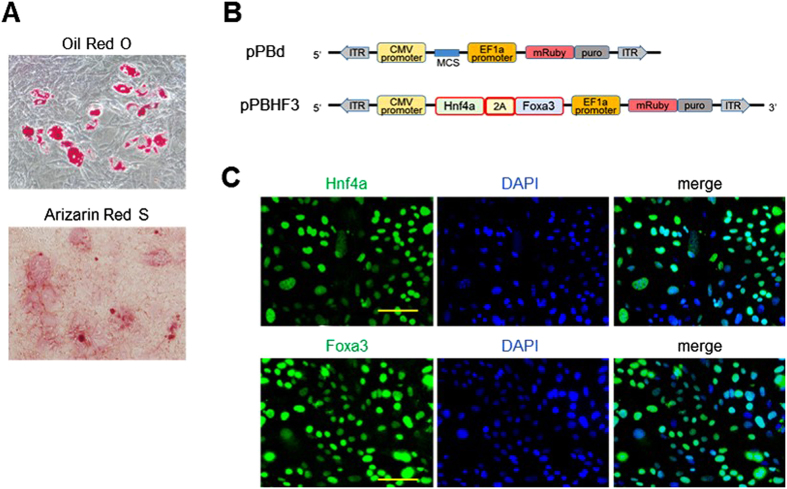
Construction of expressing vector and transfection into MSCs. (**A**) The characteristics of MSCs-derived adipocytes and osteocytes were confirmed by Oil Red O staining and Arizarin Red S staining, respectively. (**B**) pPBd possesses transposon segment indicated between inverted terminal repeat sequences (ITR), carrying CMV promoter-MCS and EF1a promoter-*mRuby*-*puro*. A set of reprogramming factors, *Hnf4a* and *Foxa3*, were linked with T2A sequence and inserted into MCS to construct pPBHF3. (**C**) pPBHF3 and pPBase were co-transfected into MSCs. After antibiotics selection, all surviving cells expressed Hnf4a and Foxa3 simultaneously. Scale bar : 100 um.

First, we confirmed the maintenance of multipotency by differentiating the mouse MSCs into adipogenic and osteogenic lineages. The lipid droplets in adipocytes were visualized using Oil Red O staining and calcified osteocytes were visualized using Alizarin Red S staining (Fig. 1A). To construct the factor-expressing vector, we selected *Hnf4a* and *Foxa3* as reprogramming factors; Suzuki *et al*.^12^ selected these factors as one of the appropriate combinations to generate iHeps, and linked them with a 2A sequence to transcribe both genes equally. Then *Hnf4a*-2A-*Foxa3* was inserted into the multi cloning site (MCS) of a dual-promoter *piggyBac* plasmid vector (pPBd), resulting in pPBHF3 (Fig. 1B). In pPBHF3, the CMV promoter was expected to drive *Hnf4a* and *Foxa3* and the EF1a promoter to drive *mRuby*-*puro*, each present within a region of the transposon segment. pPBHF3 and pPBase were co-transfected into MSCs and the cells underwent the following protocol for iHep generation; additionally, pPBd and pPBase were co-transfected into MSCs as mock-transfected samples (pPBd-TCs) and the cells underwent the same steps. Consequently, the transgene integrated into pPBHF3-transfected cells contained *Hnf4a, Foxa3 and mRuby-puro*, whereas the transgene of pPBd-TCs contained only *mRuby-puro*.

From the day after transfection, antibiotic selection with puromycin at 5 μg/ml was performed until post-transfection day 7. When the surviving cells reached confluence, all cells showed red fluorescence, suggesting that they carried the integrated transgenes. Immunofluorescent staining detected the expression of Hnf4a and Foxa3 in the nuclei of surviving cells (Fig. 1C), indicating the successful activation of the transgenes in the pPBHF3-transfected cells.

**Figure 2:**
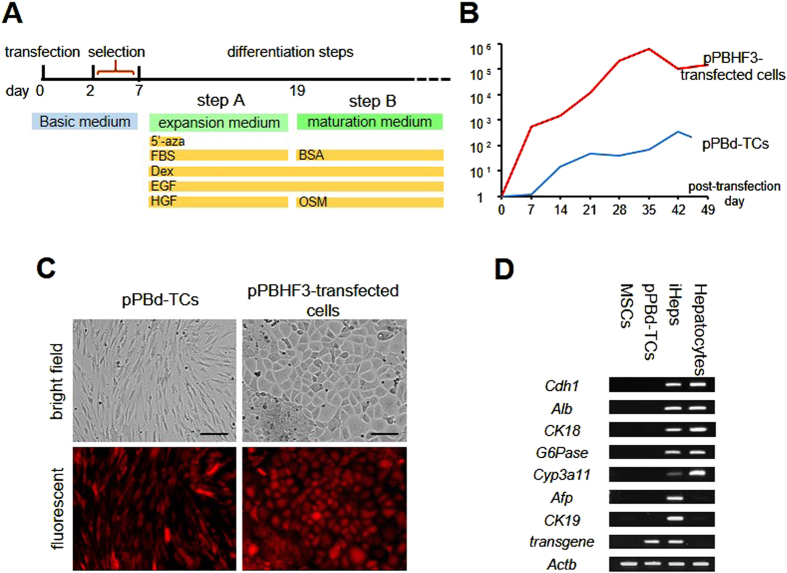
Generation of factor-integrated iHeps. (**A**) Antibiotics selection proceeded until post-transfection day 7, followed by initiation of differentiation steps. The differentiation protocol was consisted of 2 steps. Components in each medium are shown. (**B)** Albumin mRNA expression of pPBHF3-transfected cells and mock-transfected cells (pPBd-TCs) from the day of vector transfection. Differentiation step was initiated on post-transfection day7. Data were normalized to MSCs on day 0. (**C**) The morphological change in pPBHF3-transfected cells was observed on day 15 of differentiation step. All pPBHF3-transfected cells and pPBd-TCs expressed red fluorescence, indicating that transposon segments from pPBHF3 or pPBd were integrated, respectively. Scale bar: 100 um. (**D**) PCR bands of representative hepatic markers and hepatic progenitor markers are shown. Expression pattern of iHeps were similar to that of Hepatocytes but for *Afp* and *CK19* expression. iHeps and pPBd-TCs expressed bands specific to the transgene.

Cell differentiation was initiated on post-transfection day 7, beginning with step A culture in expansion medium for 12 days followed by step B culture with maturation medium (Fig. 2A). 5′-azacytidine was added for the first three days of differentiation step A. When expression of mRNA extracted every seven days was analyzed by quantitative PCR (qPCR), pPBd-TCs showed a slight but steady increase in albumin mRNA expression after post-transfection day 7, whereas pPBHF3-transfected cells showed a marked increase on day 7, with further increases thereafter (Fig. 2B). pPBHF3-transfected cells changed morphologically from a fibroblast-like shape to a polygonal shape and were densely proliferating (Fig. 2C). To evaluate reprogramming efficiency, immunofluorescent staining for E-cadherin was performed for pPBHF3-transfected cells on day 15 of the differentiation step; cells were then observed and counted using microscopy (Supplementary Fig. S1). Of the first seeded 1.34 × 10^3^ cells per field, 336 ± 8.1 fluorescence-positive cells and colonies per field were detected; therefore, the reprogramming ratio was estimated to be 25.1%. To evaluate the mRNA expression pattern, PCR products of cultured pPBHF3-transfected cells and pPBd-TCs, along with those of undifferentiated MSCs and adult mouse hepatocytes, were compared. In pPBHF3-transfected cells, representative markers typically seen in hepatocytes, such as *Cdh1* (E-cadherin), *Alb, CK18, G6Pase*, and *Cyp3a11*, were detected. Interestingly, *Afp* and *CK19*, which are hepatic progenitor markers and usually downregulated in hepatocytes, were also detected in pPBHF3-transfected cells (Fig. 2D). Thus, we considered that the pPBHF3-transfected cells had acquired hepatocyte characteristics and had been reprogrammed to iHeps.

### Characterization of transgene-integrated iHeps

**Figure 3:**
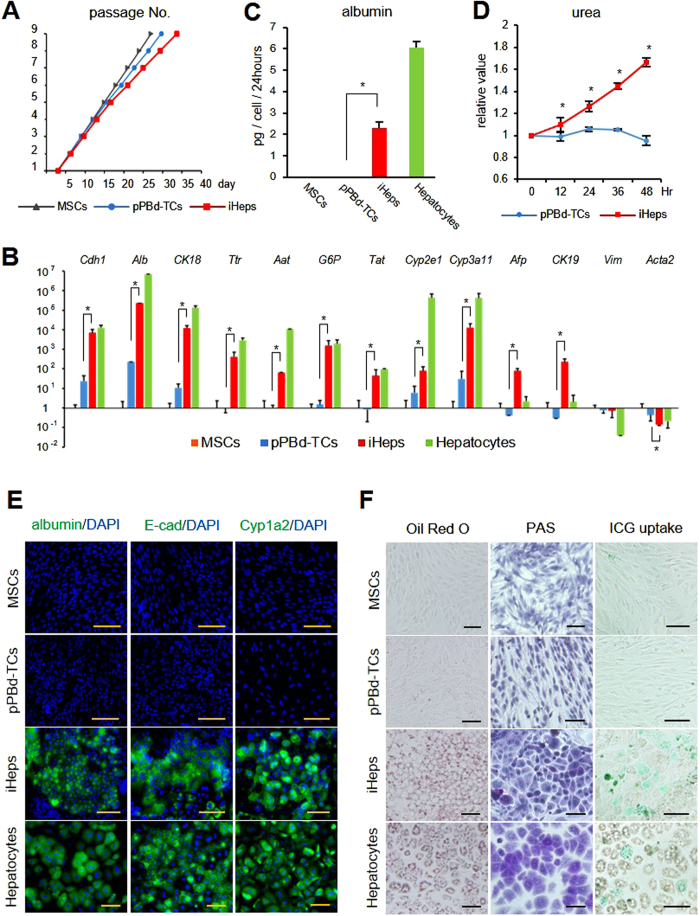
Characteristics of factor-integrated iHeps. (**A**) Each type of cells were passaged at 1:4 split when they reach confluence, until passage 9. (**B)** mRNA expression level of hepatic, hepatic progenitor and mesenchymal markers were analyzed by quantitative PCR. Data were normalized to MSCs. *p < 0.05. (**C**) Albumin secretion from each cell types were evaluated by ELISA. *p < 0.05. (**D**) Urea production of transgene-integrated iHeps and pPB-TCs were measured every 12 hours. Data were normalized to the value at 0 hour. *p < 0.05. (**E**) Immunofluorescent staining showed expression of albumin, E-cadherin and Cyp1a2 in iHeps. Scale bar: 100 um. (**F**) Storage of lipid droplets and glycogen inside the cells were evaluated by (a) Oil Red O staining and (b) PAS staining. (c) Uptake of ICG was evaluated. Scale bar: 100 um.

We next attempted to analyze the characteristics of these iHeps. To evaluate their proliferative capability, we passaged the cells at a 1:4 split ratio when they reached confluence. Although the time until confluence became gradually longer in iHeps than in pPBd-TCs and MSCs, iHeps still exhibited steady proliferation, even at passage 9 (Fig. 3A). When evaluating mRNA expression by qPCR, the levels of *Cdh1, Alb, CK18, Ttr, Aat, G6P, Tat, Cyp2e1*, and *Cyp3a11* were elevated in iHeps as in hepatocytes, and were higher than those in pPBd-TCs. Again, marked elevation of *Afp* and *CK19* was observed in iHeps. Among fibroblast markers the level of *Acta2* mRNA decreased, suggesting that transdifferentiation from mesenchymal to epithelial cells was taking place (Fig. 3B). To evaluate protein production ELISA was performed, revealing that secreted albumin was present in the culture medium of iHeps (Fig. 3C). We also evaluated urea production by measuring urea every 12 hours until 48 hours, and revealed significant increase in iHeps compared with PBd-TCs (Fig. 3D). Furthermore, immunofluorescent staining revealed the production of E-cadherin, albumin, and Cyp1a2 in iHeps (Fig. 3E). To assess mature hepatocyte function, Oil Red O staining revealed accumulation of lipid droplets, periodic acid-Schiff (PAS) staining revealed storage of glycogen, and indocyanine-green (ICG) assay showed the capability for uptake of ICG in iHeps (Fig. 3F). These mature hepatocyte functions were not observed in pPBd-TCs or in undifferentiated MSCs.

### Removal of the transposon and generation of transgene-free iHeps

**Figure 4:**
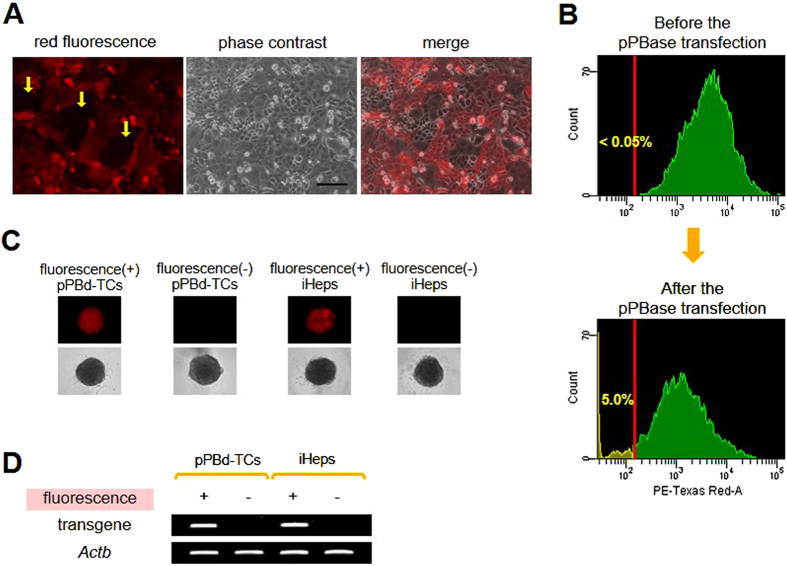
Removal of transposon from transgene-integrated iHeps. (**A**) After the transfection of pPBase into iHeps, fluorescence-negative iHeps were observed (arrow). (**B)** Majority of the iHeps which underwent pPBase transfection showed a shift toward fluorescence attenuation. The population of cells under the threshold level (red line) increased, and they were sorted. (**C**) Fluorescent and bright field observation of iHeps and mock-transfected cells (pPBd-TCs) in the form of spheroid, fluorescence(+) and (–), respectively. Scale bar: 300 um. (**D**) Removal of the transgene in fluorescence(–) spheroidal iHeps and pPBd-TCs were ascertained by lack of PCR bands with the primer specific to the transgene.

To remove the transposon segment including the transgene, the iHeps were reseeded onto dishes and transfected with pPBase alone. As a result, when the cells proliferated and reached confluence, scattered fluorescence-negative colonies were observed (Fig. 4A). Then, the cells were dispersed and analyzed using FCM. The intensity of red fluorescence was attenuated for the majority of the cells, compared to those that did not undergo pPBase transfection. An increase in the number of fluorescence-negative iHeps, for which the fluorescence intensity was under the threshold level, was observed (Fig. 4B), and these fluorescence-negative cells were sorted. pPBd-TCs underwent the same procedure. Sorted cells were then seeded onto a low-attachment 96-well plate to form spheroids (Fig. 4C). To confirm the removal of the transposon segment in these fluorescence-negative spheroidal iHeps and pPBd-TCs, PCR was performed using a primer specific to the transposon region of pPBd; a negative result indicated that transgene-free spheroidal iHeps (TFSiHeps) and pPBd-TCs (TFSpPBd-TCs) had been obtained, respectively (Fig. 4D).

**Figure 5:**
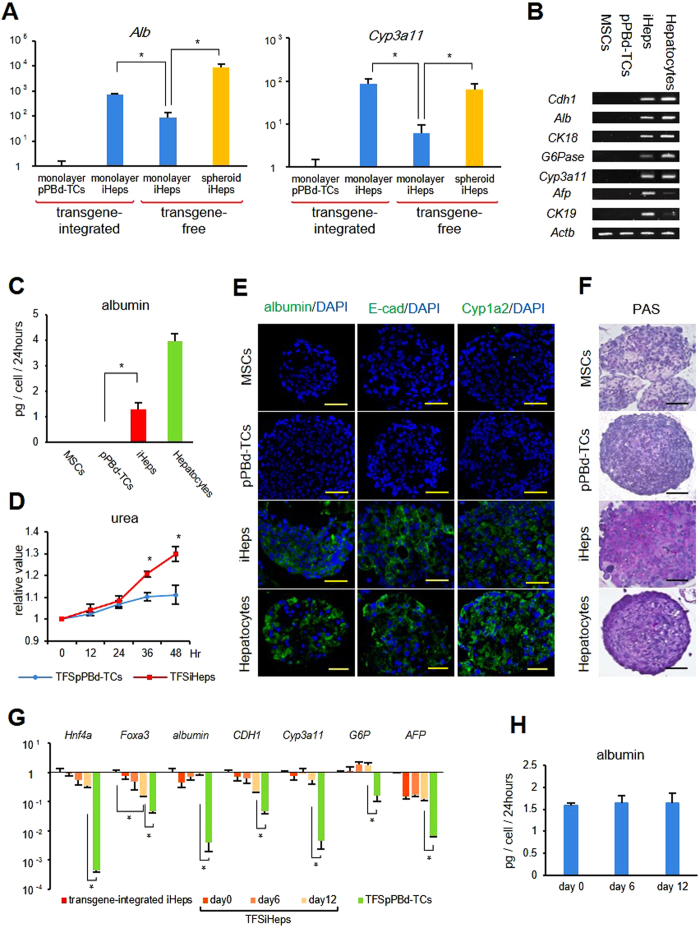
Characteristics of spheroidal transgene-free iHeps. (**A**) Quantitative PCR analysis for mRNA expression of *Alb* and *Cyp3a11*. Monolayer-cultured transgene-integrated iHeps and transgene-free iHeps, and TFSiHeps were compared with each other. Data were normalized to monolayer-cultured pPBd-TCs. *p < 0.05. (**B)** PCR bands of representative markers are shown for MSCs, transgene-free pPBd-TCs, transgene-free iHeps (TFSiHeps) and hepatocytes, each in the form of spheroid. (**C**) Albumin secretion of each spheroidal cells were evaluated by ELISA. *p < 0.05. (**D**) Urea production of TFSiHeps and TFSpPB-TCs were measured every 12 hours. Data were normalized to the value at 0 hour. *p < 0.05. (**E**) Immunofluorescent staining showed expression of albumin, E-cadherin and Cyp1a2 in TFSiHeps. Scale bar: 50 um. (**F**) PAS staining showed glycogen storage in TFSiHeps. Scale bar: 100 um. (**G**) Quantitative PCR analysis for mRNA expression of *Hnf4a, Foxa3* and other hepatocyte-specific genes. TFSiHeps which were cultured for 0, 6, 12 days after FCM were compared with monolayer-cultured transgene-integrated iHeps. Data were normalized to TFSpPBd-TCs. *p < 0.05. (**H**) Albumin secretion of TFSiHeps which were cultured for 0, 6, 12 days after FCM were evaluated by ELISA. *p < 0.05.

We then evaluated the mRNA expression level of albumin and Cyp3a11. When TFSiHeps were compared with monolayer transgene-integrated iHeps, which were cultured on dishes and did not undergo the transgene-removal process, no significant difference was observed. However, when fluorescence-negative iHeps were seeded onto dishes, the monolayer transgene-free iHeps showed lower albumin and Cyp3a11 expression levels than both monolayer transgene-integrated iHeps and TFSiHeps (Fig. 5A). Therefore, we considered that the spheroidal form was better at maintaining the hepatocyte characteristics of transgene-free iHeps. To evaluate the mRNA expression pattern, PCR products of TFSiHeps and TFSpPBd-TCs, along with those of the spheroidal undifferentiated MSCs and adult mouse hepatocytes, were compared. TFSiHeps expressed the representative markers *Cdh1, Alb, CK18, G6Pase, Cyp3a11, AFP*, and *CK19* in the same pattern observed for monolayer transgene-integrated iHeps (Fig. 5B). To evaluate protein production ELISA was performed, revealing that secreted albumin was present in the culture medium of TFSiHeps (Fig. 5C). We also evaluated urea production by measuring urea every 12 hours until 48 hours, and revealed significant increase compared with TFSpPBd-TCs (Fig. 5D). Immunofluorescent staining revealed the production of E-cadherin, albumin, and Cyp1a2 in TFSiHeps (Fig. 5E). Additionally, PAS staining revealed the storage of glycogen in TFSiHeps (Fig. 5F).

Finally, we evaluated how these hepatic characteristics change after the transgene removal at mRNA and protein level. We performed qPCR for *Hnf4a, Foxa3* and other representative hepatic markers as *Alb, Cdh1, Cyp3a11, G6Pase and AFP* (Fig. 5G), and also performed ELISA for measuring albumin (Fig. 5H) from TFSiHeps which were cultured for 0, 6, 12 days after FCM, respectively. After 12 days of culture, all genes still maintained higher expression level compared with TFSpPBd-TCs cultured for 12 days. Although a significant decrease of expression was observed in *Foxa3* compared with transgene-integrated iHeps, no significant change was observed during the culture period, indicating that expression profile was maintained after spheroid formation. No significant difference was also observed in albumin secretion between the three time points.

These results suggest that even after transgene removal, TFSiHeps maintained the same level of hepatocyte function as monolayer transgene-integrated iHeps.

## Discussion

iHeps are expected to be an alternative to hepatocytes in many situations^27^, such as a serving as a cell source for transplantation in severe liver dysfunction, *in vitro* disease modeling, pharmacokinetic analyses, and differentiation or regeneration modeling in the hepatic lineage. In the initial reports on mouse iHeps, Suzuki *et al*.^12^ transduced *Hnf4a* along with *Foxa1, Foxa2*, or *Foxa3* as reprogramming factors into mouse fibroblasts using a retrovirus, and Huang *et al*.^13^ transduced *Gata4, Hnf1a*, and *Foxa3* using a lentivirus. Both research groups confirmed that the iHeps acquired hepatic function and exhibited supportive effects on transplantation in a Fah^−/−^ mouse model. Thereafter, various research groups successfully generated human fibroblast-derived iHeps^28^,^29^,^30^,^31^, and progress also occurred in the development of culture protocols and the search for suitable reprogramming factors. However, most studies on direct reprogramming utilize integrative viral vectors. These vectors carry *in vitro* risks such as insertional mutagenesis and genotoxicity^14^,^15^ as well as *in vivo* risks such as unexpected reactivation of the transgene and virus-related toxicity^32^,^33^. Therefore, a safer reprogramming vector is essential if iHep-related cell therapy is to be applied in human medicine in the future. The *piggyBac* transposon vector system used in this study is a non-viral vector that provides efficient integration. It has several advantages such as constant gene expression as well as retention of the transgene in the transfected cells even over long culture periods or repeated passages, which should enable antibiotic selection. Owing to its suitability for reprogramming, *piggyBac* has also been applied to generate iPSCs^33^,^34^,^35^ and has recently attracted attention as an effective vector for genetic engineering^36^.

To our knowledge, this is the first report to demonstrate iHep generation using *piggyBac*-based direct reprogramming. In this study, we showed that forced expression of an integrated transgene containing *Hnf4a* and *Foxa3* successfully reprogrammed MSCs into hepatocyte-like cells. The obtained iHeps exhibited hepatocyte-specific gene expression and protein production. The iHeps also exhibited hallmarks of mature hepatocyte function, such as lipid accumulation, glycogen storage, and ICG uptake. However, the maintenance of proliferative capability and expression of hepatic progenitor markers, such as *AFP* and *CK19*, suggest that the iHeps possess the characteristics of premature hepatocytes as well.

MSCs have been investigated as a possible source of cells that can acquire hepatic characteristics when cultured in specific conditions^5^; therefore, we speculated that they would be appropriate for generating iHeps by direct reprogramming. In the present study, MSCs without overexpression of reprogramming factors were treated as mock-transfected cells and exhibited limited expression of hepatocyte-specific genes; additionally, they did not exhibit any function of maturated hepatocytes in this differentiation protocol, even in the form of spheroids. In contrast, when reprogramming factors were overexpressed in MSCs, they manifested mature hepatocyte function, indicating that efficient reprogramming was achieved.

We also demonstrated that fluorescence-negative iHeps, which were confirmed to be transgene-free iHeps, still exhibited hepatocyte function in spheroidal form comparable to that of transgene-integrated iHeps. Moreover, we showed that hepatic profile of transgene-free iHeps was maintained in the form of spheroid. Although the expression of *Foxa3* decreased as expected after the exogenous expression was lost, *Hnf4a* did not show significant decrease. The persistent endogenous expression of *Hnf4a* is considered to be one of the reasons for maintaining hepatocyte function, because Hnf4a plays a central role for the maintenance of hepatocyte differentiation^37^,^38^.

It is also suspected that the reprogrammed cells that underwent gene removal and FCM processes were susceptible to a decrease of viability and acquired hepatocyte function, whereas the cell-cell interactions in the spheroidal iHeps supported the maintenance of viability and promotion of differentiation into hepatocytes^39^,^40^,^41^. Thus, 3D culture environments including spheroids are expected to contribute to the maintenance and maturation of iHeps as reported for MSCs, iPSCs, and ESCs^42^,^43^,^44^, which could be an interesting avenue for future research. Although the present study utilized a simple model with 2 reprogramming factors, other factor combinations can also be used in the *piggyBac* system to achieve iHeps with higher levels of hepatocyte functions; as many as 6 factors have been reported^4^ (*HNF1A, HNF4A, HNF6, ATF5, PROX1*, and *CEBPA*).

Because risk-reduced transgene-free iHeps are expected to be useful for achieving therapeutic effects against liver disorder, studies to transplant these iHeps into the liver disorder models are desirable. In general, a large number of cells are required for cell transplantation, but cells do not proliferate in spheroidal form, which makes it difficult for the present model to supply abundant iHeps for cell transplantation. To overcome this problem, exploration of culture conditions that support the proliferation of transgene-free iHeps while maintaining hepatocyte functions is necessary. For example, the above mentioned 3D culture environment could serve as an alternative to spheroids, and progress in cell culture techniques with small compounds or cytokines may further support such efforts.

In conclusion, we suggest that the *piggyBac*-mediated method is a novel and promising approach to generate non-viral, transgene-free iHeps. Further advancement in achieving higher function and optimal culture conditions will facilitate the application of iHeps for upcoming cell therapies against severe liver dysfunction.

## Methods

### Preparing MSCs

C57BL/6 mouse bone marrow-derived mesenchymal stem cells (MSCs), passage 6, were purchased from Cyagen Biosciences. MSCs were seeded and expanded on collagen 1-coated dishes (AGC TECHNO GLASS) in DMEM containing 10% FBS (Invitrogen) and 1% penicillin/streptomycin (Invitrogen). MSCs of passage 8–9 were used for experiments.

MSCs underwent adipogenic and osteogenic differentiation steps using differentiation kits (Cyagen) and the protocol was carried out according to the manufacturer’s instructions. In brief, for adipogenesis, adipogenic differentiation medium was fed for three days, followed by MSC maintenance medium for a day, and repeat this process for three times. Then cells were cultured in MSC maintenance medium for seven days. Differentiated cells were evaluated by Oil Red O staining. For osteogenesis, cells were cultured in osteogenic differentiation medium for three weeks and observed after Arizarin Red S staining. MSCs were certified to exhibit a cell surface marker pattern positive for CD29, CD44, CD31 and Sca-1 and negative for CD117 by flow cytometry (FCM) by manufacturer.

### Vector construction

*PiggyBac* dual promotor transposon vector (pPBd) along with *piggyBac* transposase expressing vector (pPBase) were purchased from System Biosciences. pPBd has multi-cloning site (MCS) down stream of CMV promotor, and *mRuby* and *puro* down stream of EF1a promotor.

*Hnf4a* and *Foxa3* cDNA were obtained from mouse total mRNA by reverse transcription PCR. Then both of them were linked together via T2A sequence by recombinant PCR to construct *Hnf4a*-2A-*Foxa3* segment, adding *Xba1* and *BamH1* sequence on each end. This segment was inserted into *Xba1*/*BamH1* restriction site among MCS of pPBd by digestion and ligation, resulting in pPB-Hnf4a-2A-Foxa3-mRubyPuro (pPBHF3).

### Cell culture/generation of transgene-integrated iHeps

pPBHF3 and pPBase were co-transfected into MSCs with Lipofectamine 3000 (Invitrogen). pPBd and pPBase were transfected into MSCs in the same manner as mock samples (pPBd-TCs). After medium was changed, puromycin was added for antibiotic selection and when cells reached confluent the differentiation step was initiated.

For the first twelve days, as step A, cells were cultured in expansion Medium consisted of DMEM/F-12 (Invitrogen) containing 10% fetal bovine serum (FBS) (Invitrogen), 1% penicillin/streptomycin (Wako), 10 mM nicotinamide (Sigma Aldrich), 100 μM 2-melcaptomethanol (Wako), 100 μM ascorbic acid (Wako), 1 μM Dexamethasone (Dex) (Wako) and ITS (BD Biosciences), supplemented with 20 ng/ml hepatocyte growth factor (HGF) (R&D Systems) and 20 ng/ml epidermal growth factor (EGF) (R&D systems). Then step B was initiated with maturation medium consisted of IMEM + GlutaMax (Invitrogen) containing 0.5% bovine serum albumin (BSA) (Wako), 1% penicillin/streptomycin, 10 mM nicotinamide, 100 μM 2-melcaptomethanol, 500 μM ascorbic acid, 1 μM Dex and ITS, supplemented with 20 ng/ml oncostatin M (OSM) (R&D Systems) and 20 ng/ml EGF. For the first three days of the differentiation step, 200 uM 5′-azacytidine (5′-aza) was added to expansion medium.

### Generation of transgene-free iHeps

pPBase alone were transfected into iHeps with ViaFect (Promega) and cells were expanded until confluence for a few days. Then iHeps which were negative for red fluorescence were sorted by FCM, detected in PE-Texas Red filter. We prepared cells as described previously. The threshold level for red fluorescence positive was determined as the level under which less than 0.05% of the transgene-integrated cells, which did not undergo transgene-removal process, remained. Sorted cells were reseeded onto low attachment 96well V bottom plate (SUMILON) at 1 × 10^4^ cells per well to form spheroids. After 1 week of culture, TFSiHeps along with TFSpPBd-TCs were collected for mRNA extraction, staining and ELISA, and culture medium for urea measurements. The image of whole protocol is depicted in Supplementary Fig. S2.

### Primary hepatocyte isolation and culture

Adult C57BL/6 mice were subjected to two-step collagenase perfusion for isolation of primary hepatocytes as described previously^4^,^45^. Briefly, after laparotomy portal veins were cannulated with 25-gauge needles. Through these needles, the livers were preperfused with 10 ml Hanks-EGTA. Subsequently, the livers were perfused with 15 ml collagenase solution containing 0.3% dispase II (Sanko Junyaku) and 0.3% collagenase II (Gibco). Then the livers were minced with a scissor and filtered thorough 50 N polypropylene mesh, and the suspension was centrifuged two times at 50 g for 4 min. Cell pellets were resuspended and cultured with HBM (Lonza) onto collagen 1-coated dish for two days for RNA extraction or other experiments. For spheroid formation cells were seeded into 96well V bottom plate and cultured for four days until analyses.

### PCR/Quantitative PCR

PCR and RT-PCR assays were performed as previously described^46^,^47^,^48^. Total RNA was extracted with PureLink RNA mini kit (Thermo Fischer Scientific) and then reverse-transcribed into cDNA with Revertra Ace (Toyobo). qPCR was performed with Power SYBR Green (Applied Biosystems). The quantification of the expression of genes were evaluated as the relative level normalized to endogenous Actin beta. Primer sequences for PCR analyses are listed in Supplementary Table S1.

### Immunofluorescent staining

The cultured cells were fixed and stained as previously described^49^. Primary antibodies were as follows; mouse anti-human Hnf4a (PPMX), goat anti-human Foxa3 (Santa Cruz), goat anti-mouse albumin antibody (Bethyl Laboratories), mouse anti-mouse E-cadherin (Abcam), mouse anti-mouse cytchromeP450 1A2 (Abcam) at 1:200 respectively. The sections were incubated for two hours with secondary antibodies; Alexa 488-conjugated rabbit anti-goat IgG or goat anti-mouse IgG at 1:1000. After washing, the stained sections were covered with Vectashield mounting medium with DAPI (Vector Laboratories).

Spheroids were first embedded in iPGel (NIPPON Genetics) and fixed with 4% PFA overnight, then embedded in paraffin and cut in 5 μm thick. After deparaffinization and antigen retrieval using a Target Retrieval Solution (Dako), nonspecific binding was blocked with 1% bovine serum albumin (Sigma Aldrich) dissolved in 0.1% Polyoxyethylene Octylphenyl Ether (Wako) in PBS. The primary and secondary antibodies reactions were carried out in the same way for cultured cells.

### Measurements of albumin and urea

Cells on 35 mm collagen1-coated dishes and spheroids in 96 well plate were incubated in newly fed maturation medium, and culture supernatants were collected. For albumin measurement, culture medium for 24 hour was collected. The quantity of albumin was calculated using mouse albumin ELISA kit (Bethyl Labolatories) and urea using QuantiChrom Urea Assay Kit (BioAssay Systems), each according to the manufacturer’s instructions.

### Oil Red O staining, Periodic Acid-Schiff (PAS) staining, ICG uptake assay

For Oil Red O staining, linoleic acid (Wako) was added into the medium at 10 mg/ml and incubated for 30 minutes. After washed with PBS, cells were fixed with 4% PFA at room temperature for 30 minutes and washed with 60% isopropanol, followed by incubation for 30 minutes with Oil Red solution which Oil Red powder (Sigma Aldrich) was dissolved into isopropanol and diluted to 60% with distilled water. Then cells were washed with distilled water three times. For PAS staining, cultured cells were stained by periodic acid-Schiff (MUTO PURE CHEMICALS) following the manufacturer’s instructions. For paraffin-embedded spheroids, the same procedure was carried out after deparaffinization. For ICG uptake assay, indocyanine green (Wako) was added in the medium at 0.1 mg/ml and incubated for 1 hour, followed by washing with PBS three times.

### Statistics

The results are expressed as mean ± SEM. Statistical significance was assessed using unpaired t-test; p-values < 0.05 were considered statistically significant. Data analysis was performed by GraphPad Prism. For all statistics, data from at least three independent samples were used.

## Additional Information

**How to cite this article**: Katayama, H. *et al*. Generation of non-viral, transgene-free hepatocyte like cells with *piggyBac* transposon. *Sci. Rep.* **7**, 44498; doi: 10.1038/srep44498 (2017).

**Publisher's note:** Springer Nature remains neutral with regard to jurisdictional claims in published maps and institutional affiliations.

## Supplementary Material

Supplementary Information
