# Supplementary material for: Generation of non-viral, transgene-free hepatocyte like cells with piggyBac transposon
Source: Sci Rep. 2017 Mar 15;7:44498. doi: 10.1038/srep44498 (PMC5353749; doi:10.1038/srep44498)
Supplement: Supplementary Information [file srep44498-s1.pdf]

Generation of non-viral, transgene-free hepatocyte like cells with *piggyBac* transposon.

Hokahiro Katayama[1], Kentaro Yasuchika[1,*], Yuya Miyauchi[1], Hidenobu Kojima[1], Ryoya Yamaoka[1], Takayuki Kawai[1], Elena Yukie Yoshitoshi[1,2], Satoshi Ogiso[1], Sadahiko Kita[1], Katsutaro Yasuda[1,2], Naoya Sasaki[1], Ken Fukumitsu[1], Junji Komori[1], Takamichi Ishii[1] and Shinji Uemoto[1]

[1]Department of Surgery, Graduate School of Medicine, Kyoto University, Kyoto, Japan
[2]Center for iPS Cell Research and Application (CiRA), Kyoto University, Kyoto, Japan

S1

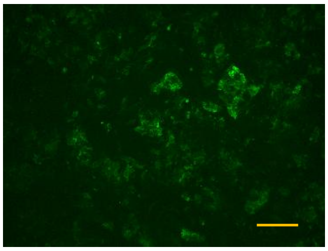

S2

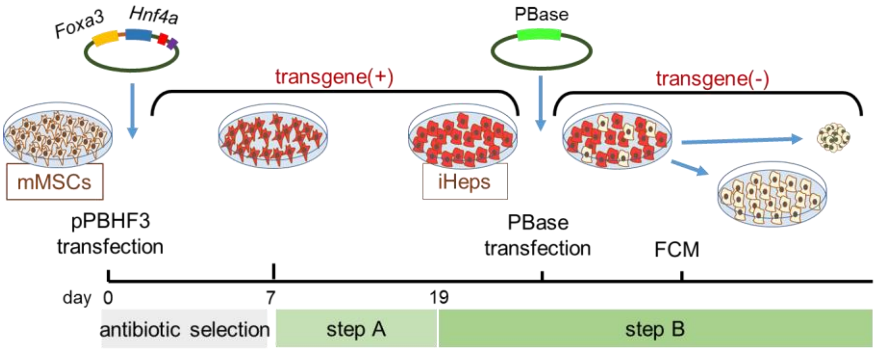

Supplementary Figure. (**S1**) Immunofluorescent staining for E-cadherin on day 15 of differentiation step. Scale bar : 200um. (**S2)** Schematic image of whole protocol is described.

	**forward**	**reverse**
Aat	TTGACGTGCACTATTGCAGC	TGTTCCTCTCTCATCGATGG
Actb	AGGGAAATCGTGCGTGACAT	GCTAGGAGCCAGAGCAGTAATC
Afp	ACAGGAGGCTATGCATCACC	TGGACATCTTCACCATGTGG
Alb	GACAAGGAAAGCTGCCTGAC	TTCTGCAAAGTCAGCATTGG
Acta2	CTATTCAGGCTGTGCTGTCC	AGTTCGTAGCTCTTCTCCAGG
CK18	AAGATCATGGCGGACATCC	CTGACTCCAGATGCAGAAGG
CK19	GTGCCACCATTGACAACTCC	AATCCACCTCCACACTGACC
Cyp2e1	TGGTCCTGCATGGCTACAAG	CGGGCCTCATTACCCTGTTT
Cyp3a11	AGCAGGGATGGACCTGGTTT	CAGCAAGGAGAGGCGTTTGA
Cdh1	GCCACAGATGATGGTTCACC	CTTCATGCAGTTGTTGACCG
G6P	AAGTCGTTCCCATTCCGCTT	CAAAGGGAACTGTTGCGCTC
Tat	TCCAGGAGTTCTGTGAACAGC	AGTATATGGTGCCTGCCTGC
Ttr	ATGAATTCGCGGATGTGG	TTCCTGAGCTGCTAACACGG
Vim	TACCAGGACACTATTGGCCG	CTGTTGCACCAAGTGTGTGC
**transgene**	TGAGGGCAGACATAGCACAG	AGTTCTTGCAGCTCGGTGAC

Supplementary Table. (**S1**) Primers for PCR analyses are listed.
